# Role of Fungicides, Application of Nozzle Types, and the Resistance Level of Wheat Varieties in the Control of *Fusarium* Head Blight and Deoxynivalenol

**DOI:** 10.3390/toxins3111453

**Published:** 2011-11-16

**Authors:** Ákos Mesterházy, Beáta Tóth, Monika Varga, Tibor Bartók, Ágnes Szabó-Hevér, László Farády, Szabolcs Lehoczki-Krsjak

**Affiliations:** 1 Cereal Research Non-profit Company, H-6701 Szeged, P.O. Box 391, Hungary; Email: beata.toth@gabonakutato.hu (B.T.); vargam@gabonakutato.hu (M.V.); tibor.bartok@fumizol.hu (T.B.); agnes.szabo@gabonakutato.hu (Á.S.-H.); lehoczkisz@gabonakutato.hu (S.L.-K.); 2 Bayer Hungaria Ltd., Alkotas Str. 50, 1123 Budapest, Hungary; Email: laszlo.farady@bayer.com

**Keywords:** *Gibberellazeae*, *F.culmorum*, scab, AUDPC, FDK, DON, prothioconazole, tebuconazole, carbendazim

## Abstract

Fungicide application is a key factor in the control of mycotoxin contamination in the harvested wheat grain. However, the practical results are often disappointing. In 2000-2004, 2006-2008 and 2007 and 2008, three experiments were made to test the efficacy of fungicide control on *Fusarium* Head Blight (FHB) in wheat and to find ways to improve control of the disease and toxin contamination. In a testing system we have used for 20 years, tebuconazole and tebuconazole + prothioconazole fungicides regularly reduced symptoms by about 80% with a correlating reduction in toxin contamination. Averages across the years normally show a correlation of *r* = 0.90 or higher. The stability differences (measured by the stability index) between the poorest and the best fungicides are about 10 or more times, differing slightly in mycotoxin accumulation, FHB index (severity) and *Fusarium* damaged kernels (FDK). The weak fungicides, like carbendazim, were effective only when no epidemic occurred or epidemic severity was at a very low level. Similar fungicide effects were seen on wheat cultivars which varied in FHB resistance. In this study, we found three fold differences in susceptibility to FHB between highly susceptible and moderately resistant cultivars when treated with fungicides. In the moderately resistant cultivars, about 50% of the fungicide treatments lowered the DON level below the regulatory limit. In the most susceptible cultivars, all fungicides failed to reduce mycotoxin levels low enough for grain acceptance, in spite of the fact that disease was significantly reduced. The results correlated well with the results of the large-scale field tests of fungicide application at the time of natural infection. The Turbo FloodJet nozzle reduced FHB incidence and DON contamination when compared to the TeeJet XR nozzle. Overall, the data suggest that significant decreases in FHB incidence and deoxynivalenol contamination in field situations are possible with proper fungicide applications. Additionally, small plot tests can be used to evaluate the quality of the field disease and toxin production.

## 1. Introduction

*Fusarium* head blight (FHB), regarded as one of the most important diseases of wheat, is caused by a number of *Fusarium* species. *F. graminearum* Schw. [teleomorph: *Gibberella zeae* Schw. (Petch.)] predominates in most parts of the world [1] while *F. culmorum* Sacc. and other *Fusarium* spp. are of rather regional and local importance [2,3]. The toxin contamination produced by the pathogens has made this disease one of the most important food safety challenges [4]. Disease resistance of wheat cultivars is the best solution; however, breeding takes a long time and highly resistant cultivars are not yet available. After the FHB epidemics in the second half of the 20th century, more than ten years were necessary to bring moderately resistant varieties to the market [5]. However, many more years will be needed before the majority of the wheat acreage is planted with cultivars with high or very high resistance. Accordingly, fungicide applications are considered as a potential solution for the present problems. As the European Union (EU) [6] has limited the DON contamination in raw grain at 1.25 mg kg^−1^, and the United States (U.S.) to 2.0 mg kg^−1^, this strict limit for DON will influence the export markets and therefore will have global consequences. 

A significant part of the literature on fungicide application is in Congress Proceedings and Extension papers which are not easily accessible but have been included in the present work. Although fungicides have been applied to manage the disease, experimental results generally indicate unsatisfactory efficacy of 50% or lower [3,7,8,9,10,11]. While some fungicides did not reduce FHB symptoms or DON levels in the grain [12,13,14], the literature suggests that tebuconazole, metconazole, prothioconazole, and bromuconazole are the most effective compounds [9,10,11,15,16,17,18,19]. However, in the years with FHB epidemics, it was seldom possible to reduce the ratio of visibly scabby to all grains to less than 5%, a percentage which was considered necessary for successful practical control [4]. In Hungary, the official limit is even less, at only 2%. Extensive tests [3] showed that fungicides containing tebuconazole (T) had the largest effect, but efficacy depended on the concentration used. The most effective was Folicur (T250) at an application rate of 1 L/ha with 250 g/L active ingredient per hectare (a.i./ha). 

### 1.1. Fungicides

In the last decade, a new fungicide, prothioconazole (P), has appeared on the market. Like other triazoles, P is a demethylation inhibitor, with broad effects against a very wide spectrum of fungal diseases [17]. Suty-Heinze and Dutzmann [20] reported that it also has good efficacy against FHB in wheat. The reduction of the mycotoxin levels was 58-60% for a mix of P125 + T125 as compared with 43-48% for T250 alone. Testing of this fungicide (P) has been very intensive in the U.S. under the company code AMS 24619 and in Europe under the company code JAU 6476. Reports in different tests indicated a reduction in disease and/or DON levels ranging from 22-72% [8,19,21,22,23,24]. T250 alone exhibited significantly lower efficacy than P250 alone. All other fungicides tested were less effective. 

Comparison of data from fungicide studies is often difficult due to differences in locations (environmental factors), disease severity (high to low), type of fungicide used (alone and in combinations), cultivars of wheat (spring, winter), method of inoculation (natural, corn cob inoculum, spray inoculum), and method of study (misting, bagging of heads, data collection). In spite of these variables, Uniform Fungicide Trials (UFTs) have been established in the U.S. and conclusions can be made based on the data obtained. Prothioconazole is often cited as being the most reliable fungicide in reducing DON levels in both winter and spring wheat [7,25]. In order to predict whether grain from a field will have high or low levels of DON, correlation coefficients were analyzed between DON and disease indicators from 163 individual studies [26] including the US, Europe, Canada, and Africa, involving both spring and winter wheat. *Fusarium* damaged kernels (FDK) showed the strongest correlation with DON levels with a mean of *r* = 0.73, while the correlation with head severity was only *r* = 0.52. We also see similar correlations, but at times the visual symptoms gave closer correlations than did FDK [3,27,28]. Even if FDK is most closely correlated with DON levels, measurement of FDK still involves threshing the seed and counting individual seeds for disease, a time-consuming process and not easily done in the field.

One of the most widely advocated and tested products for FHB in the U.S. is tebuconazole (Folicur) and numerous Uniform Fungicide Trials have included this compound. A meta-analysis was used to assay the effect of fungicides, including tebuconazole, on FHB and DON content in wheat grains [26]. The researchers found that tebuconazole was more effective at limiting disease severity (mean proportion of diseased spikelets per spike) than at limiting DON levels and the efficacy was also greater in spring wheat than winter wheat. However, the researchers conclude that the decision to spray wheat fields with tebuconazole must include such monetary factors as the cost of the application *versus* the increased income from any increase in higher quality grain. The decision to use any fungicide should include other management practices, such as tillage, crop rotation, and using resistant cultivars. Another in-depth study of triazole-based fungicides concluded that P125 + T125 was the most effective fungicide for limiting disease severity while metconazole was the most effective treatment for reducing DON levels [26]. Work continues on testing different fungicide formulations [29] and testing fungicides on moderately and susceptible wheat cultivars [4], but fungicide use may be sufficient to reduce DON contamination during a weak epidemic but not when the FHB epidemic is strong. This means that fungicides may not provide sufficient control when it is most needed.

### 1.2. Application of Fungicides

As stated by Paul *et al.* [30], more critical studies are needed to evaluate the efficacy of the fungicides when used as part of an integrated management program to ascertain the overall percent control of disease and DON production. The timing of the application of the fungicide is important for FHB control [3]. Blandino *et al.* [31] have found approximately 48% DON reduction following fungicide application (prochloraz, T and tebuconazole-azoxystrobin) at mid-anthesis (Feekes scale 10.52-10.53). Schneider *et al.* [32] found that early or late spraying was less effective than at anthesis. Blandino *et al.* [31] reported that a double treatment, with application of strobilurine prior to flowering and application of triazole during flowering, resulted in DON reduction.

Studies involving different wheat cultivars have shown that fungicide treatment of cultivars that are moderately susceptible (MS) or moderately resistant (MR) to FHB may not reduce DON levels [33]. However, other studies have shown that more resistant cultivars provide higher fungicide efficacy, and while the susceptible cultivars show improved fungicide efficacy, the improvement is not always sufficient for a satisfactory reduction of DON [3,4,10,34]. For this reason, research is necessary to form a comprehensive view of the problem.

Triazole fungicides appear to be only partially systemic in wheat. They are distributed more or less evenly in the sprayed organ, e.g., leaf or glume, but they do not translocate well from the leaves to the heads or from one part of the head to another [35]. The triazoles also have some growth regulatory effects based on their cytokinin-like activity [36,37,38,39]. Tests of present technologies showed that coverage of heads by fungicides is very low and uneven. McMullen *et al.* [40] found significant differences in coverage and distribution of spray on heads. Halley *et al.* [41] evaluated several spraying technologies and found that the back of the head seldom received more than 10% coverage, and the front normally about 20%. Hooker and Schaafsma [42] demonstrated that the traditional and newer spraying technologies generally give low coverage, normally not better than 10%, while aerial application gave only 1-3%. However, the Turbo FloodJet nozzle gave uniform coverage above 30% on each side [42]. Ruden *et al.* [43,44] found that the deep penetration of the spray into the heads down to the rachis is as important as coverage of the outer surface of the head. These data clearly indicate that poor coverage may be a major cause of the generally poor efficacy experienced in many fungicide trials. For these reasons, we felt that farm scale testing of better nozzle types was very important. 

During the past 20 years, we have used a hand spraying method that has given optimal coverage by spraying from the side [3,10]. This method concentrates on good coverage and has shown a reduction of symptoms and an average reduction in DON contamination of 80% when using tebuconazole. For the present study, the effect of spraying was analyzed using artificial inoculation with four isolates of *Fusarium* spp. on three cultivars of wheat with different levels of resistance, and with two different nozzles for fungicide application.

### 1.3. Stability of Fungicide Performance

A myriad of studies using fungicides are available that provide useful information to scientific researchers and the general public [26,29,30,45]. However, it also is important to have information on fungicide stability, similar to what is part of the plant breeding practice [46,47]. For example, using the plant breeding method, Mesterházy [27] evaluated the stability of FHB resistance expression on 25 wheat genotypes. However, to our knowledge, there is no literature on the stability evaluation of fungicides on FHB. We therefore chose to check the general stability of the fungicides with regard to all FHB epidemic situations. Although early studies reported increased FHB severity following powdery mildew or other leaf diseases [48], we do not have evidence that fungicide control of these foliar disease results in a reduction of FHB. We therefore chose to include this study in the research program. The FHB resistance level is significantly influenced by the success of the fungicide treatment. Wheat varieties also influence fungicide effect but in a more complicated way. Along with level of resistance and flowering type [49], we have added fungicide receptivity. When cultivars have the same resistance level, but highly differing response to fungicides, the differences may be due to morphology traits such as the presence of awns that can catch additional spray, plant height that influences the landing of spray on the head, and other such things.

### 1.4. Objectives

The objectives of the present study were:

1. evaluate the efficacies of fungicides on FHB and their influence on DON concentrations using different strains of *Fusarium* and different cultivars of wheat.
2. assay stability of the fungicide performance across different epidemic situations.
3. evaluate fungicide performance on pre-flowering and flowering plants.
4. compare results from small plot treatments with farm scale treatments using different methods of fungicide application.

## 2. Materials and Methods

### 2.1. Experimental Design

For Experiments 1 and 2, winter wheat plots were planted following oil rape (*Brassica napus*) in order to minimize inoculum from debris remaining from the previous crop. The plot size was 5 × 1 m and the wheat was sown at 550 seeds/m^2^. Experiment 1 (Results Section 3.1, 3.2) plots were sown on October 28, 25, 15, 18 and 22 in 2000, 2001, 2002, 2003, and 2004, respectively, using a Wintersteiger Øyord planter (Wintersteiger GmbH, Ried, Austria). Three winter wheat cultivars with different levels of resistance to FHB were planted: Zugoly (susceptible, S), Sámán (moderately susceptible, MS) and Bence (moderately resistant, MR). Experiment 2 plots (Results Section 3.3) were planted in 2007 and 2008. Two varieties (Petur MR and Samson MR) were used. Sowing time was on 19 and 23 October.

For experiments 1 and 2, seeds were sown in a randomized block design in three plot replicates. Within each plot, groups of about 20 heads were selected as subplots for the artificial inoculation treatment. Treatments included four isolates of *Fusarium* spp. in three replicates (side by side 50, 100, 150 and 200 cm from the plot front) and the non-inoculated control (50 cm from the back side of the plot). Additionally, we included a treatment without fungicide use as a *Fusarium* check; here only the *Fusarium* inoculation was applied. Each *Fusarium* treatment was evaluated in three subplots providing a mixture of factorial and complete block design. 

Experiment 3 (Results Section 3.4) compared results between small plots (design as in Exp. 1) and farm scale level made in 2006-2008 with Petur (MR), Miska (SS) and Kapos (S). For large plot testing, the cultivars were sown in 250 m long × 135 m wide strips, 250 × 400 m plots, in a 10 ha field. The fungicide treatments were made across the strips at full flowering, with two nozzles, using Turbo FloodJet and TeeJet XR nozzles. The fungicides were evaluated across nozzles and cultivars (6 sets of data) and nozzles were rated across fungicides and cultivars (27 sets of data). The Turbo FloodJet was chosen as Hooker and Schaafsma [42] found this nozzle gave much better coverage than traditional nozzles developed for the control of leaf diseases. TeeJet XR is the most used nozzle in Hungary and this served as a control. The boom was 17 m wide, two m under the tractor was not treated, and the left and right part was 7 m. The 17 × 135 m plot was divided into four subplots giving four possibilities for nozzle tests on a plot size of 7 × 67.5 m. Fungicide treatments were made at full flowering. The nozzle size was chosen for a spray volume to provide 250 L/ha at a speed of 7-8 km/h. To evaluate coverage, two methods were applied. (1) A UV sensitive color was mixed with the fungicide and sprayed on the plants with different nozzles. Several days later, heads were collected and marked for front side and the percent coverage was assessed under UV light (Tungsram Hungary (General Electric), Budapest, type: F10T8BL, UV tube, UV-A spectrum, 350-365 nm). Twenty ears were separately analyzed for each type of nozzle application. (2) Water sensitive paper strips were mounted on sticks at head level, and after spraying, the coverage (%) was evaluated with the aid of a computer (image analyzer). In Experiment 3, the plots were not artificially inoculated with *Fusarium* strains so FHB was caused by naturally occurring *Fusarium* strains and subjected to natural environmental conditions. 

The location of experimental fields are several hundred meters from the GPS coordinates: 46°11'42.15"; 20°8'56.13". The field is in the Tisza river valley, it is alluvial with medium to high clay content, it has high humus content (3-4%), and deep production depth of about 1 m with excellent water economy. Fertilization was given at 60 + 60 + 60 kg a.i. for NPK (nitrogen, phosphorus, potassium) in the autumn before plowing (mid-September) and an additional 60 kg N was given at the beginning of April. The yield of grain each year also depends on the amount of rainfall, with the yearly means of grain between 4 and 10 t/ha. 

### 2.2. Fungicide Application

Fungicides (Table 1 and Table 2) were applied at Feekes growth stage [1] 10.51, from the beginning of flowering to within 2-3 days, with 0.5 L hand sprayers fitted with graduated flasks. In cooler seasons, two spraying times were necessary. In warmer springs, only one application was used. Each fungicide was applied in 250 mL of water per 5 m^2^ plot (500 L/ha), with half applied from each side of the plot so that the heads were thoroughly covered. The dosages agreed with recommendations from the manufacturers. All the three small plot fungicides tests were made using optimal coverage. 

**Table 1:** Fungicides and rates 2000-2008.

Experiment	Commercial Name and Rate L/ha	
	Leaves	Heads
**1**	No	Prosaro 1.0
	No	Folicur 1.0
**2000-2004**	No	Falcon 0.8
	No	Kolfugo Super 1.5
	UTC	UTC
**Experiment**	**Commercial Name and Rate L/ha**	
	**Leaves**	**Heads**
**2**	Falcon 0.6	Prosaro 0.8
	Nativo 1.0	Prosaro 1.0
	UTC	Folicur Solo 1.0
	UTC	Juwel TT 1.2
	Nativo 1.0	Falcon 0.8
**2007-2008**	Acanto 0.75 + Talius 0.15	Alert S 1.0
	Tango Star 0.8	Juwel TT 1.2
	UTC	Falcon 0.8
	UTC	Artea 0.5
	Amistar Xtra 0.8	Artea 0.5
**Experiment**	**Commercial Name and Rate L/ha**	
	**Leaves**	**Heads**
**3**	UTC	UTC
	No	Prospekt 1.5
	No	Falcon 0.8
	No	Prosaro 1.0
**2006-2008**	No	Tango Star 1.0
	No	Eminent 1.0
	No	AmistarXtra 1.0
	No	Juwel 1.0
	No	Artea 0.5
	No	UTC

**Table 2:** Active ingredients of the fungicides and their abbreviations.

Commercial Name	Active Ingredient (a.i.) g/L	Abbreviation. of a.i. and Rates
Acanto	pikoxystrobin 250	PIK200
Alert S	fluzilazole 125 + carbendazim 250	Flu125 + C250
AmistarXtra	azoxystrobin 200 + ciproconazole 80	AX200 + CC80
Artea	propiconazole 250 + ciproconazole 80	Pro125 + CC40
Eminent	tetraconazole 125	TET125
Falcon	tebuconazole 133 + spiroxamine 250 + triadimenol 43	T133
Folicur Solo	tebuconazole 250	T250
Juwel	epoxyconazole 125 + kresoxym-methyl 125	EP125 + K125
Juwel TT	epoxyconazole 83 + kresoxym-methyl 83 + fenpropimorf 317	EP100 + K100 + F380
Kolfugo Super	carbendazim 200	C300
Nativo	tebuconazole 200 + trifloxystrobin 100	T200 + TR100
Prosaro	Prothioconazole 125, tebuconazole 125	P125 + T125
Prosaro 0.8	Prothioconazole 125, tebuconazole 125	P100 + T100
Prospekt	Carbendazim 200, propiconazole 80	C300 + P120
Talius	proquinazid 200	PQ30
Tango Star	epoxyconazole 84 + fenpropimorf 250	EP84 + F250

### 2.3. Inoculum Production

For each test in a year, four epidemic situations were used generated by different isolates. As resistance background to *F. graminearum* and *F. culmorum* is the same [28,50] and the fungicide reaction to these two species is the same [3], the epidemic situations can be analyzed together without problem. 

For Experiment 1, two isolates of *Fusarium graminearum*, 12377 (from maize seed; Vesztő, Hungary, 1978) and No. 44 (from wheat grains; Tulln, Austria, 1992) and two *F. culmorum* isolates, 12375 (wheat root; Szeged, Hungary, 1978) and 12551 (wheat stalk base; Szeged, Hungary, 1978), were used. For Experiment 2, the isolates used in 2007 were: *F. graminearum* 12377 and K2P1, the latter was isolated from naturally infected fields in Kiszombor in 2006, and *F. culmorum* 12375 and 12551. Isolates used for 2008 were: two inocula from *F. culmorum* 12551, A and B, and two inocula from *F. graminearum* 12377, A and B. For Experiment 3, the isolates used in 2006 and 2007 were: *F. graminearum* 12377 and J5A2/A (the latter from Kiszombor 2005), and *F. culmorum* 12375 and 12551. The conidium concentrations and aggressiveness levels are given in Table 3. All *F. graminearum* isolates belonged to *F. graminearum* stricto senso of the *F. graminearum* complex (51). The *F. culmorum* isolates belonged to a Hungarian group that was separate from the western European and American groups [52], yet the virulence was similar in both groups [53]. Aggressiveness of the inocula of all isolates was confirmed prior to testing using bioassays in Petri dishes [27,54,55]. Isolates were stored in test tubes on potato dextrose agar under light mineral oil (Soltrol 160) at room temperature. Isolates stored under these conditions remained viable for 3 years and did not lose their aggressiveness [56], but the aggressiveness of their inocula did vary from year to year.

**Figure 1:**
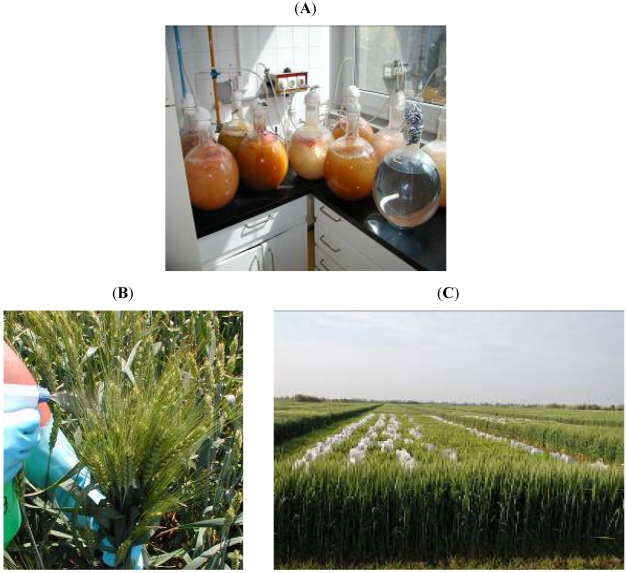
(A) Inoculum production in 10 L glass balloons on liquid Czapek-Dox medium; (B) Inoculation with *Fusarium* suspension; (C) Experiment showing bagged heads.

The inocula were prepared by the bubble-breeding method [56], shown in Figure 1A. The concentrations of conidia were measured with a Buerker cell-counting chamber. The inocula were stored at 4°C until use. 

**Table 3:** Conidium concentration (×10^6^) of the isolates and their aggressiveness, 2000-2008.

Isolate	2000		2001		2002		2003		2004	
Exp. 1.	CFU/mL ^1^	Aggr. (%) ^2^	CFU/mL	Aggr. (%)	CFU/mL	Aggr. (%)	CFU/mL	Aggr. (%)	CFU/mL	Aggr. (%)
12551 *F. culmorum*	0.27	90.0	0.00 M *	56.0	0.10	38.0	0.32	90.0	0.55	72.5
12375 *F. culmorum*	0.00 M	76.0	0.05	75.0	0.16	100.0	0.15	100.0	0.05	74.0
44 *F. graminearum*	0.18	89.0	0.03	53.0	0.00 M	100.0	0.12	100.0	0.00 M	22.5
12377 *F. graminearum*	0.05	75.0	0.76	74.5	0.17	68.0	0.43	100.0	0.12	5.0
**Isolate**	**2006**		**2007**		**2008**					
**Exp. 2. **	**CFU/mL**	**Aggr. (%)**	**CFU/mL**	**Aggr. (%)**	**CFU/mL**	**Aggr. (%)**				
12551 *F. culmorum*	0.3	45.0	0.15	32.0	0.03	91.0				
12375 *F. culmorum*	0.00 M	83.0	0.00M	85.0	0.10	95.0				
44 *F. graminearum*	0.00 M	59.0	0.13	17.0						
12377 *F. graminearum*	0.0	45.0	0.02	21.0	0.05	60.0				
46.06/2 *F. graminearum*					0M	80.0				
**Isolate**	**2007**		**2008**							
**Exp. 3.**	**CFU/mL**	**Aggr. (%)**	**CFU/mL**	**Aggr. (%)**						
12551 *F. culmorum*	0.02	52.0	0.03	74.0						
12375 *F. culmorum*	0.05	73.0	0.37	81.0						
12377 *F. graminearum*	0.01	42.0	0.35	58.0						
12377 *F. graminearum*	0.88	86.0								
46.06/2 *F. graminearum*			0,00 M	80.0						

^1^ concentration of conidia ×10^6^. Mycelial fragments present in the suspension that were not counted; ^2^ Aggressiveness: Mean of diseased germinating wheat seeds across five readings (2–6 days after starting the test) and across four dilution rates (original, 1:1, 1:2 and 1:4) related to the non-inoculated controls in two lines with differing germling resistance, M* mycelium occurred.

### 2.4. Inoculation

Two to three days after fungicide treatment (Feekes growth stage 10.51), arbitrarily chosen groups of approximately 20 flowering heads were sprayed from all sides with the inoculum (about 20 mL for each group) using a 1 L hand sprayer (Figure 1B). This was normally done between 20 and 28 May in the different years. Heads of border rows were not used [3]. After inoculation, the heads were covered with a polyethylene bag for 48 h (Figure 1C). Non-inoculated control spikes were sprayed with distilled water and bagged in the same way at the end of the plot. No additional mist irrigation was applied.

As indicated previously, each of the four fungal isolates was applied in triplicate within each main plot (5 m^2^). Each group of heads was labeled and loosely bound until harvest.

### 2.5. Evaluation of Disease and Toxin Analysis

Head symptoms were evaluated at 10, 14, 18, 22, and 26 days after inoculation by estimating the percentage of the diseased spikelets of the group of heads tested, *i.e.*, the severity of the disease [56] and called FHB. Leaf rust (*Puccinia* spp.) and leaf blotch (*Septoria tritici*) were also observed as well as minor amounts of powdery mildew and wheat tan spot (*Drechslera tritici-repentis*).

At harvest, the heads of each subplot were cut and put in separate paper bags. Ten random heads were separated from each bag. They were threshed by a Wintersteiger Seed Boy thresher without wind to retain all of the grains, even severely shriveled kernels [3]. Chaff was removed using an air separator Ets Plaut-Aubry (41290 Conan-Oucques, France). Wind speed was finely regulated to keep all grains. The grains were evaluated for FDK by estimating the ratio of scabby grains as a percentage of total grains while in white plastic triangle dishes. For DON analyses, three samples of grain from an isolate from each plot were pooled [27]. Six grams of grain were milled, of which 5 g was used for DON extraction and HPLC analysis using an HP 1090M equipped with diode array detector. The method of DON analysis is given in Mesterházy *et al.* [3].

In testing the application of fungicides using different spray nozzles, visual symptoms were evaluated as incidence, being the number of visually infected heads counted in 10 sample areas of 1 m^2^, within each plot of 7 × 67.5 m. From the yield of each plot, two subsamples were separated and were subjected to FDK visual score and DON analysis. 

### 2.6. Statistical Analysis and Stability Determination

Stability of fungicide efficacy was determined by calculating the slope of the linear regression line from the means of a fungicide treatment (Y axis) plotted against the appropriate corresponding means for fungicide non-treated control (X axis) [46]. For each fungicide, the cultivar × *Fusarium* isolate means (*i.e.*, data average across subplots and plot replicates) were used to calculate the regression line. This provided 12 data epidemic situations (three cultivars and four isolates) for each year and in the five years altogether 60 data points were considered. DON levels for T125 + P125 treatments were only measured four years, resulting in 48 data points. A low slope indicates that a fungicide treatment provided a high level of control across mild to severe epidemics. A high slope indicates that the fungicide did not provide sufficient control during medium or high epidemic severity. 

For all three small plot tests, the randomized block design was combined with nested-factorial design [3]. First, the means were produced for all isolates within a plot and these data served as entries into the ANOVA. Data were analyzed together for the whole experimental period allowing analysis for fungicides, cultivars, isolates, years and their interactions. The severity of FHB, ratio of FDK, and the DON values of the non-inoculated control groups of heads were zero or near zero in all tests; these data therefore were not included in the analyses. In all tests, the plots that were inoculated with *Fusarium* without fungicide treatment served as controls to calculate efficacy or reduction. 

The data of the five to six readings (depending on the year) for FHB were averaged first for each subplot, as mean data better express the amount of the disease. Then the three subplot means were averaged again for individual isolates and these means served as entries to the ANOVA. So every entry represents 15 or 18 field data points. For FDK, the data of the three subplots were averaged for an isolate and these mean data were the entries to the ANOVA. For DON analysis, the grains of the three subplots were pooled and from them an analysis for DON was made and this was the entry for the ANOVA. The efficacy was calculated for each entry compared with the corresponding data of the fungicide-free *Fusarium* inoculated control. ANOVAs were calculated for all efficacy data, and their least significant difference (LSD) values are also given. 

In Exp. 1, the same isolates were used in all five years, so an isolate effect in the ANOVA could be determined. In tests 2 and 3, the isolates changed, therefore epidemic situations were analyzed.

Correlation and regression analyses were made with the built-in programs of Microsoft Excel. The four-way analyses were conducted via Microsoft Excel with the functions given by Sváb [57] and Weber [58]. Comparing the slopes for significance, the functions from Sváb [57] were used. In several cases, the statistical program SPSS (SPSS Hungary 1115 Budapest Bartók Béla street 105-113) was used. 

## 3. Results

### 3.1. Effect of Fungicides on FHB

From the data from years 2000-2004 where four fungicide treatments were selected (T125 + P125, T250, T133, and C300), to test against four *Fusarium* isolates (44Fg, 12377Fc, 12375Fc, and 12551Fc), on three different wheat cultivars (Zugoly-FHB susceptible, Sámán-FHB MS, and Bence-FHB MR), the visual disease assessment data had a similar pattern to FDK (Table 4) and DON (Table 5). P125 + T125 showed 0.87% visual infection severity, T250 showed 1.51%, T133 showed 2.23%, while the fungicide-free control was at 17.5%, on average of the three cultivars. The least significant difference at 5% (LSD 5%) was 0.59, *i.e.*, all fungicides differed in their capacity to decrease FHB. However, the T125 + P125 treatment was significantly more effective than tebuconazole alone. In efficacy or reduction of symptoms (severity of disease) the numbers were: 95, 91, 87 and 47%, for P125 + T125, T250, T133 and C300, respectively. The LSD 5% was 1.7%. The efficacy varies between 89 and 98% for the best fungicides in the different epidemic situations, and 9.8-63% for the least effective (carbendazim). The three cultivars differed in response, as the best efficacy was measured on the most susceptible cultivar, Zugoly, and the least efficacy on the more resistant cultivars. 

In the analysis of the overall mean effect of fungicides on FDK values (Table 4), the non-sprayed and inoculated controls had 25.2% FDK, P125 + T125 had 2.1%, T250 had 4.3%, T133 had 5.6%, and the C300 fungicide had 12.58% across years, isolates and cultivars. Accordingly, the reduction was high, and we saw 91.7% efficacy for P125 + T125, 82.9% efficacy for T250, 77.6% efficacy for T133, and only 49.2% for C300. The LSD 5% value was 3.6%. Interestingly, for the more resistant cultivar Bence, the efficacy data for almost all fungicides were lower than that found for the more *Fusarium* susceptible cultivars of Sámán and Zugoly. The data varied much less for the most effective than the least effective fungicide. All fungicides differed significantly from each other.

**Table 4:** Effect of fungicides against FHB in wheat: *Fusarium* damaged kernels (FDK) (% of scabby grains) in three cultivars tested during 2000-2004.

Fungicides	Zugoly (S)					Sámán (MS)					Bence (MR)				
a.i. g/ha	44Fg	12377Fg	12375Fc	12551Fc	Mean	44Fg	12377Fg	12375Fc	12551Fc	Mean	44Fg	12377Fg	12375Fc	12551Fc	Mean
P*125 + T125.	2.4	0.6	0.4	3.3	**1.7**	1.5	1.1	1.2	7.4	**2.8**	1.7	1.0	1.2	3.6	**1.9**
T250	7.3	3.6	2.3	7.2	**5.1**	2.6	1.7	1.0	11.4	**4.2**	3.1	1.1	2.6	7.9	**3.7**
T133	8.2	2.4	1.7	13.4	**6.4**	8.8	3.7	2.5	13.5	**7.1**	2.8	1.0	2.4	7.4	**3.4**
C300	22.3	13.5	13.1	28.8	**19.4**	13.6	4.9	6.8	17.2	**10.6**	5.7	4.2	5.9	17.5	**8.3**
*Fusarium* check	39.2	30.6	32.7	39.2	**35.4**	31.6	17.9	15.3	39.6	**26.1**	16.3	7.7	11.7	20.9	**14.1**

* T = tebuconazole, P = prothioconazole, C = carbendazim.

**Table 5:** Effect of fungicides against FHB in wheat. DON contamination in mg kg^−1^ on three cultivars during 2000-2004.

Fungicides	Zugoly (S)							Sámán (MS)					Bence (MR)		
a.i. g/ha	44Fg	12377Fg	12375Fc	12551Fc	Mean	44Fg	12377Fg	12375Fc	12551Fc	Mean	44Fg	12377Fg	12375Fc	12551Fc	Mean
P*125 + T125.	2.0	0.8	0.9	2.7	**1.6**	1.2	0.3	0.4	3.3	**1.3**	2.2	0.7	0.8	1.6	**1.3**
T250	4.4	2.2	1.4	2.7	**2.7**	2.6	1.4	0.7	6.6	**2.8**	5.5	1.1	2.4	3.8	**3.2**
T133	6.5	1.5	1.6	6.8	**4.1**	4.2	2.0	1.5	7.2	**3.7**	4.0	0.8	1.9	3.1	**2.4**
C300	8.9	5.6	6.5	13.3	**8.6**	9.1	1.5	3.3	9.5	**5.9**	5.9	1.8	3.8	7.3	**4.7**
UTC + *Fusarium*	21.6	12.5	32.7	27.1	**23.4**	26.0	25.4	13.2	24.7	**22.3**	13.6	5.1	7.1	13.9	**9.9**

* T = tebuconazole, P = prothioconazole, C = carbendazim.

The DON level data (Table 5) are the most important as acceptability of grain is based on whether DON levels are under the food safety limit for tolerable level. The P125 + T125 treatment lowered the toxin contamination below the acceptable limit in seven cases, according to European standards, and in nine cases for the U.S. standard. For T250 treatment, two cases met the European standards, and four the U.S. standard. The difference between P125 + T125 and T250 treatments is significant. T133 reduced the DON level in 1 (European std.) and 6 (U.S. std.) cases , and carbendazim reduced the DON level below the U.S. in only 2 cases. The efficacies of fungicides on DON reduction showed that P125 + T125 varied between 84.1% and 98.7%, depending on the strain of fungus and the cultivar of wheat, with an overall mean of 92.4%. T250 gave slightly better results than T133, but the difference was not significant. C300 results varied from 46.2 and 94% with a mean 65.6%. As with the results from the severity of disease and the FDK analysis, fungicide efficacy on DON reduction was greater in the more FHB susceptible wheat cultivars (82-85%) than on the more resistant cultivar (66%). 

The yield data showed the same tendencies as we saw for FDK and DON, but the efficacies were much lower (data not shown). 

An ANOVA presents the mean square (MS) values for the analyses (Table 6) to show any and all main effects and interactions of the fungicides on FHB occurrence, FDK, and DON, when considering the variables of the years, the wheat cultivars, and the fungal isolates. It is apparent that the fungicide treatment has the most significant effect on FHB, FDK, and DON, regardless of the year, the fungal isolate used, or the wheat cultivar. So the fungicide activity can be reproduced well under very different epidemiological conditions. 

**Table 6:** Mean Square (MS) values for ANOVAs of the traits tested in the fungicide trials 2000-2004.

Source of Variance	df	MS Values			
		FHB	FDK	df (DON)	DON
Fungicide A	4	9192.3 ***	33659.3 ***	**4**	6977.1 ***
Year B	4	1271.1 ***	933.3 ***	3	1587.9 ***
Isolate C	3	1857.1 ***	12161.8 ***	3	879.9 ***
Cultivar D	2	381.9 ***	3369.5 ***	2	934.6 ***
AxB	16	403.3 ***	**102.3 ns**	**12**	556.3 ***
AxC	12	484.0 ***	2410.4 ***	**12**	**46.3 ns**
AxD	8	320.4 ***	2722.5 ***	**8**	502.3 ***
BxC	12	1160.4 ***	2316.5 ***	9	1527.9 ***
BxD	8	1891.5 ***	1639.8 ***	6	712.3 ***
CxD	6	96.4 ***	4404.6 ***	6	127.6 ***
AxBxC	48	240.2 ***	688.0 **	**36**	279.5 ***
AxBxD	32	431.1 ***	536.5 **	**24**	841.6 ***
AxCxD	24	30.5 **	696.1 **	**24**	132.7 ***
BxCxD	24	362.0 ***	1414.9 ***	18	250.5 ***
AxBxCxD	96	90.7 ***	825.0 ***	**72**	146.4 ***
Within	600	16.2	297.9	480	25.3

Bold: Fungicide main effect and interactions with fungicides. *** *P* = 0.001, ** *P* = 0.01, ns = non-significant, significance according to *F* test.

The correlation coefficient between FHB and FDK is *r* = 0.9671, for FHB-DON, *r* = 0.9521, and for FDK-DON, *r* = 0.9656; all are significant at *P* = 0.001. The very close correlations tell clearly that the fungicide effect decreases not only the FHB symptoms but also the other measured parameters like DON and FDK. Of course, when individual data are examined and not the general means, the correlations are less close. For example, a correlation between FDK and DON was *r* = 0.7697, *n* = 725, significant at *P* = 0.001. This means that the correlation, even if it is reduced by 0.2, is close enough to have good predictive value in the given experiment. However, this does not mean that FDK can predict the levels of DON produced under different conditions. Therefore, an exact analytical method is needed to verify the quantity of toxin contamination in all cases in question.

### 3.2. Stability Tests

For a fungicide to be useful for any farmer, it should be effective under any environmental conditions, upon any wheat cultivar, and against any *Fusarium* species. Our experimental conditions were set up for just such a stability analysis. We found that the lowest slope of the regression analyses for FHB was *b* = 0.064 under the fungicidal treatment of P125 + T125. For T250 the slope was 0.1010, for T133 it was 0.131, and for C300 it was 0.6199. The stability for this trait in P125 + T125 was ten times better than for C300. For FDK the best slope was 0.09 for P125 + T125 while the worst was 0.606 for C300. This is again a 10-fold difference between slopes. For DON, P125 + T125 had a slope of 0.019 and C300 had 0.182, again a 10-fold difference.

The FDK data from the 60 individual epidemic situations clearly show that fungicide treatment using P125 + T125 was usually highly effective, however, in some cases it produced only moderate control. Again, C300 was not very effective when there was a high disease level in the non-treated check.

**Figure 2:**
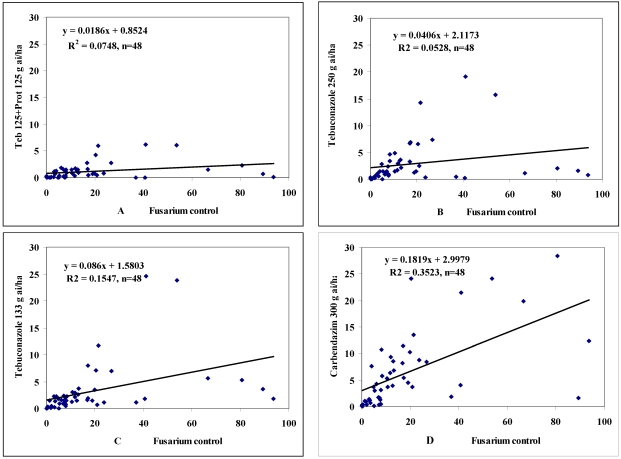
Stability of fungicides for controlling deoxynivalenol (DON) contamination, data: mg kg^−1^. Data: four years *three cultivars* four isolates (=48 epidemic situations). Data of the *Fusarium* inoculated but not fungicide treated controls (X axis) were plotted against the data of the four fungicides tested (Y axis). (Commercial names: (A) Prosaro; (B) Folicur; (C) Falcon; (D) Kolfugo).

The stability data for FDK was very similar to that of DON. When disease severity was high and DON levels of up to 10 mg kg^−1^ were detected in the *Fusarium* check, fungicide treatments of P125 + T125 satisfactorily controlled the levels of DON (Figure 2). When individual samples were analyzed, several samples from fungicide treated heads surpassed the 2 mg kg^−1^ value, but there were many more examples for excellent control. When DON levels above 10 mg kg^−1^ were detected in the *Fusarium* checks, the fungicide control was not able to reduce the DON levels below the 2 mg kg^−1^ level, even though the reduction may have exceeded 80-90%. Tebuconazole 250 had lower performance, with maximum values of DON in fungicide-controlled samples of 20 mg kg^−1^, which was four times more than the P125 + T125 treatment. For T133, the maximum level of DON was 25 mg kg^−1^, however, at lower epidemic severities, the control was satisfactory. Carbendazim (C300) gave satisfactory control for DON contamination only in cases of low disease severity; otherwise the control was far from sufficient.

### 3.3. Influence of Leaf Diseases on the Success of Head Control

It is possible that diseases of the leaf of wheat are sufficient to increase the susceptibility of the head to FHB. To test this, we applied an early fungicide spray at the two node development stage followed by a head treatment at flowering. The DON data show that at low epidemic severity, levels of DON at 0.5-3 mg kg^−1^ were reduced to 0.2-0.4 mg kg^−1^ by the best fungicides. In cases with high levels of DON, 22 mg kg^−1^ in the controls were reduced to 5 mg kg^−1^, a nearly 80% reduction, however, still higher than the 1.25 mg kg^−1^ that is acceptable. The general means of the three traits, FHB, FDK and DON (Table 7) showed that, in general, leaf control did not significantly decrease FHB symptoms when compared to head treatment. The main conclusion is that leaf diseases do not appear to be related to FHB severity. The treatments, however, increased plot yield for each fungicide between 2% (Pro125 + CC40 head, and UTC for leaf) and 11% (P125 + T125 head, and T200 + TR100 for leaf). The plot yield for T133 (0.4%) increased due to the leaf treatment by T200 + T100 significantly by 8.4% . The yield difference between EP100 + K100 + F380 head treatment and the influence of the leaf treatment of EP67 + F200 did not change, both were 1.58 and 1.9% higher than the control, but there was no significant difference between them. The same was true for Pro125 + CC40 that increased yield by 5.16%, while the leaf treatment had an additional yield increase of 0.9%, which was not significant.

**Table 7:** Summary table for the disease traits in the fungicide tests on leaves and/or heads across years (2007 and 2008) and epidemic situations. A correlation of the traits is given at the bottom.

Fungicides		FHB	FDK	DON	Reduction			
Leaves Rate g/ha	Head Rate g/ha	%	%	mg kg^−1^	FHB%	FDK%	DON%	Mean
T100	P100 + T100	0.34	0.45	1.17	95.31	93.99	79.05	89.45
T200 + TR100	P125 + T125	0.73	0.89	1.34	89.90	88.27	76.12	84.77
UTC	T250	0.56	1.28	1.92	92.28	83.05	65.73	80.35
PIK200 + PQ30	FLU + C	2.27	2.22	2.02	68.53	70.63	63.94	67.70
T200 + TR100	T133	1.46	2.14	2.03	79.76	71.71	63.71	71.72
UTC	T133	1.65	2.60	2.67	77.06	65.53	52.26	64.95
UTC	EP100 + K100 + F380	1.98	1.99	2.67	72.51	73.59	52.33	66.15
EP67 + F200	EP100 + K100 + F380	2.14	2.60	3.04	70.26	65.54	45.66	60.49
UTC	Pro125 + CC40	4.43	6.10	4.35	38.45	19.20	22.40	26.68
AX160 + CC64	Pro125 + CC40	4.81	6.34	5.33	33.22	15.99	4.90	18.04
UTC	UTC	7.20	7.55	5.60				
Correlation of traits		FHB	FDK	DON				
FDK		0.9744						
DON		0.9530	0.9780	1				

All significant at *P* = 0.001.

### 3.4. Small and Large Plot Comparisons

In small plot experiments run during 2006-2008, FHB data showed a significant reduction of symptom severity when fungicides were applied (data not shown). For the more resistant cultivar, Petur, the best fungicide (P125 + T125) reduced FHB severity to 1% or lower compared to the untreated controls which ranged from 2.9% (low disease) to 33% (high disease). The moderately sensitive cultivar Kapos also had reduced FHB levels that were near the Hungarian regulatory levels of 2% or lower even under medium to high levels of disease when P125 + T125 was used (check range 4.7-34%). When P125 + T125 was applied to the susceptible cultivar Miska, the FHB values were 4-8% compared to the check value 15-60%. This reflected an eight fold higher disease level than the mean values of Petur under the same fungicide treatment. The FDK values from the small plot analyses presented a similar picture. At high epidemic severity, the FDK levels in Petur were decreased from 53% in the controls to 2.3%, while in Miska, the levels were decreased from 66% in the controls to 10%, when using P125 + T125 treatment. The less effective fungicides, e.g., Pro125 + CC40 and TET125, showed much lower reduction in FDK values (data not shown). 

**Table 8:** Small plot fungicide control of *Fusarium* head blight in wheat. DON contamination mg kg^−1^ during two low, one medium, and one high epidemic.

Fungicide	Petur (MR)						Miska (S)						Kapos (MS)					
	Low	Low	Medium	High	UTC	Mean	Low	Low	Medium	High	UTC	Mean	Low	Low	Medium	High	UTC	Mean
P125 + T125	0.19	1.74	1.96	7.65	0.38	**2.38**	2.29	2.25	8.64	7.90	0.42	**4.30**	1.44	1.95	9.29	5.75	1.30	**3.95**
T133	0.63	1.19	3.31	9.62	0.40	**3.03**	11.63	9.22	29.63	33.92	1.87	**17.25**	8.10	11.07	63.17	45.07	0.81	**25.64**
P125 + T125	3.09	1.25	8.75	16.53	0.57	**6.04**	13.24	7.57	35.86	40.30	0.81	**19.56**	6.56	10.03	45.66	38.60	4.09	**20.99**
T133	1.54	0.85	4.96	18.78	0.51	**5.33**	9.30	10.62	36.73	42.93	1.69	**20.26**	11.26	12.89	55.39	38.42	0.75	**23.74**
EP125 + K125	2.56	1.70	11.00	19.55	0.39	**7.04**	10.98	8.29	41.24	35.80	1.06	**19.47**	8.43	12.82	43.99	51.44	1.44	**23.62**
EP84 + F250	3.99	3.52	12.13	21.48	0.89	**8.40**	14.91	10.77	30.10	45.67	1.16	**20.52**	9.39	14.47	49.01	44.93	1.07	**23.77**
Pro125 + CC40	4.11	2.83	14.31	26.07	0.98	**9.66**	15.08	7.44	43.51	59.22	1.75	**25.40**	5.44	12.65	64.74	54.37	1.12	**27.66**
TET125	4.32	3.82	15.02	26.15	1.34	**10.13**	13.24	11.59	49.36	55.41	1.36	**26.19**	8.27	13.57	75.36	68.47	1.38	**33.41**
UTC + *Fusarium*	4.54	2.15	13.07	41.44	1.14	**12.47**	19.84	14.33	74.88	73.92	2.30	**37.06**	13.30	10.74	111.78	70.49	1.12	**41.49**

The data on the DON levels are perhaps the most important (Table 8) as trade is regulated by this trait. It seems that an acceptable fungicide control is not possible when natural infections cause a DON level above 10 mg kg^−1^. For an example, using the MR cultivar Petur under environmental conditions that produced high levels of disease, DON concentrations averaged 41 mg kg^−1^ in the untreated controls while P125 + T125-treated wheat had DON concentrations averaging 8 mg kg^−1^. While the reduction in DON levels was 80%, the remaining levels were still too high to be acceptable for trade. Although FDK and DON levels had a correlation of *r* = 0.81 at *n* = 108, significant at *P* = 0.001, we found that a direct forecasting of the DON via FDK was not possible. We found, for example, an FDK level of 0.11% had a DON level of 1.74 mg kg^−1^ DON, while an FDK level of 0.54% had a DON level of 3.52 mg kg^−1^. Samples with an FDK level lower than 2% contained DON levels from 0.19–3.99 mg kg^−1^. An FDK of 20% may occur with a DON contamination between 7 and 62 mg kg^−1^. The variety resistance actually influences the success of the fungicide significantly. On the more resistant cultivar Petur, all fungicides performed better than on the more susceptible cultivars. The data show, however, that if the DON level is not higher than about 10 mg kg^−1^ in the non-sprayed and *Fusarium* infected control, fungicide treatment can decrease the DON level to the legal limit or lower.

There was a significant influence of environmental conditions on the 3 year small plot/large farm plot experiments. 2006 and 2007 were rather dry, and the DON levels were about 50% less than the FDK levels. In the very wet year of 2008 the situation changed and there were high levels of disease. The amount of FHB and FDK doubled from the mean of 2006 and 2007, while the DON contamination increased 10-fold (Table 9). This latter was highly sensitive to late rains.

**Table 9:** Comparison of FHB, FDK and DON values in the small plot fungicide tests 2006-2008. Data: means across isolates, fungicides and cultivars.

Year	FHB Severity%	%FDK	DON mg kg^−1^
2006	7.63	9.22	3.82
2007	3.99	8.69	4.93
2008	13.75	19.63	44.44
Mean	8.46	12.51	17.73

It is important to determine whether a fungicide has similar efficacy on various traits (FHB, FDK, DON) under different environmental conditions. We found that, once again, P125 + T125 was the best treatment with 88% reduction in all traits measured during the 3 year study. ANOVA showed results very similar to that of the Table 5 of Exp. 1 (results/data not shown). 

The FHB incidence data of the farm scale experiment (Table 10) shows clear fungicide differences on the different cultivars using different spray nozzles for fungicide application. As the farm plots were not artificially inoculated with *Fusarium* strains, the level of disease was caused only by naturally occurring *Fusarium* strains and subjected to natural environmental conditions. This resulted in relatively low levels of FHB over the 3 years. The *Fusarium* check controls showed FHB incidence of 6 infected heads/m^2^ for cultivar Petur, 10 infected heads/m^2^ for cultivar Kapos, and 16 heads/m^2^ for cultivar Miska. The best fungicide, Prosaro 1.0 (P125 + T125), reduced the FHB incidence by 90% or more regardless of the resistance of the cultivar or the type of nozzle used. However, the Turbo FloodJet nozzle consistently reduced the FHB incidence better than the TeeJet XR nozzle, with the percentage decrease differing for each cultivar and for each fungicide. The ANOVA showed highly significant fungicide and nozzle differences at *P* = 0.001. 

**Table 10:** FHB incidence (infected head/m^2^) of the farm scale fungicide test across years 2006-2008.

Treatment	Petur		Miska		Kapos	
	Teejet XR	Turbo FloodJet	TeeJet XR	Turbo FloodJet	TeeJet XR	Turbo FloodJet
P125 + T125	0.37	0.07	2.13	1.00	1.37	0.93
T133	0.63	0.30	5.77	3.63	3.67	2.13
C300 + P120	4.37	0.70	6.23	4.47	4.70	2.70
EP125 + K125	1.30	0.97	7.33	5.37	5.00	3.60
EP84 + F250	2.43	1.00	8.67	6.17	6.97	3.10
Pro125 + CC40	1.70	1.53	8.60	6.87	6.23	4.80
AX200 + CC80	2.23	1.47	9.37	6.63	7.17	5.57
TET125	2.57	1.07	13.33	11.00	8.33	6.43
UTC *Fusarium* natural	5.87	5.87	15.73	15.73	10.40	10.40

**Table 11:** DON (mgkg^−1^)data of the farm scale tests of FHB control by fungicides in wheat, 2006-2008.

Treatment	Petur		Miska		Kapos	
	TeeJet XR	Turbo FloodJet	TeeJet XR	Turbo FloodJet	TeeJet XR	Turbo FloodJet
P125 + T125	0.06	0.00	0.63	0.31	0.33	0.40
T133	0.22	0.06	1.08	0.90	0.74	0.00
C300 + P120	0.23	0.07	1.35	0.58	0.73	0.34
AX200 + CC80)	0.31	0.08	0.89	0.61	1.09	0.58
EP125 + K125)	0.06	0.06	1.42	0.72	1.48	0.67
Pro125 + CC40	0.22	0.09	1.65	0.65	1.37	0.75
EP84 + F250	0.19	0.00	1.40	0.97	1.73	0.60
TET125	0.14	0.08	1.07	1.23	2.10	0.35
UTC	0.43	0.43	1.84	1.84	1.02	1.02

Although the FDK numbers were low in the *Fusarium* checks and there was no significant difference in FDK between the TeeJet XR or Turbo FloodJet nozzle applications, the fungicides that were applied did have differing effects on FDK. The most effective fungicides, P125 + T125 and T133, reduced FDK numbers by 79 and 84% respectively. The levels of DON detected in the grain (Table 11) showed a very similar picture to what we have seen before. In the more resistant cultivar Petur, the untreated control (UTC) had 0.43 mg kg^−1^ DON, far under the EU limit of 1.25 mg kg^−1^, and treatment by any fungicide, regardless of the nozzle type of application, reduced the DON levels below the UTC. In the sensitive cultivar of Miska, the checks showed 1.84 mg kg^−1^, and the traditional nozzle, although lowering the DON levels slightly, could not consistently reduce DON levels to under 1.25 mg kg^−1^ while the Turbo FloodJet mozzle could. In cultivar Kapos, the UTC had DON levels of 1.0, and the DON levels for various fungicide treatments varied. The TeeJet XR nozzle did not reduce the DON levels as much as did the Turbo FloodJet, except for the P125 + T125 treatment which had the same levels of DON. Overall, the Turbo FloodJet nozzle provided generally better reduction in DON than the TeeJet XR nozzle. 

**Figure 3:**
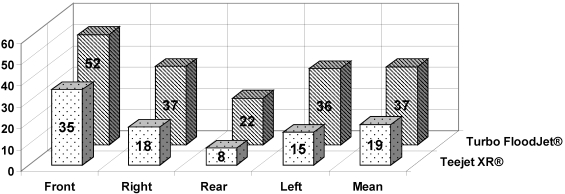
Coverage of the ears by fungicides using different nozzle types as measured by water sensitive paper stripes across three cultivars, 2007-2008.

Use of the water sensitive strips showed the type of coverage on the wheat heads by each type of nozzle (Figure 3). Use of the TeeJet XR showed that the front of the head had the best coverage (35%), the sides less, and the rear, the lowest value (8%). Use of the Turbo TeeJet resulted in a significant increase in coverage, as the front had 52% while the rear had 22%. In the UV light test, the mean coverage of the TeeJet XR was 12% while the mean coverage by the Turbo FloodJet was 27%. 

**Figure 4:**
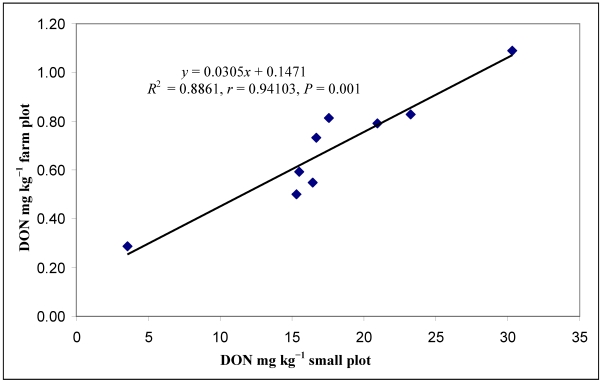
DON data (mg kg^−1^) for the fungicides in the small plot artificial and farm scale natural general means across all variables, (*n* = 9), 2006-2008.

The DON data from the artificial small plot and natural farm scale tests were also compared using the general means of the fungicides, which show the basic trend. As the results were similar to the different traits, we present only the DON data (Figure 4). The correlation between the two series is 0.94, significant at *P* = 0.001. P125 + T125 had 88% reduction in DON in the small plot test, while the farm scale test showed 73%. The least effective fungicide caused only 23% reduction in both experimental versions. The small plot efficacy data correlated with the large scale trial data, differing by only about 10%. A comparison between the DON data from artificial infection and that from natural infection from the various fungicides and across cultivars showed more variation with a correlation coefficient of *r* = 0.7079 (*n* = 27, *P* = 0.001).

## 4. Discussion

### 4.1. Fungicide Stability and Efficacy on FHB Severity

In previous reports, the fungicide efficacy values reported were generally lower than 50% even for the best fungicides [4,29,30,35,45,59,60,61,62,63]. Despite this, the use of fungicides may reduce DON during high epidemic conditions [34]. However, the food safety standard of 2 (US) or 1.25 mg kg^−1^ (EU) DON cannot be reached in most cases where fungicides are used. Paul *et al.* [45] stressed that at this low level of efficacy, the justification of the fungicide application may be questioned. and that the FHB problem needs more critical studies to better understand the system we face and to find useful solutions for the farmers. 

The results from this paper and earlier reports [3,10] show that fungicides T250 and P125 + T125 provide significantly higher reduction of FHB disease than other fungicides, as the mean data of reduction were seldom lower than 80% in the small plot tests. However, 40-50% reduction was observed also for the least effective fungicides. This means that the fungicides are much more effective than is commonly shown from the field results from large scale or small plot tests. For this reason, perhaps the statement of Paul *et al.* [45] questioning the use of fungicides needs some reconsideration. From these numbers we propose that there is a technology gap. The technology gap is the difference in full coverage of wheat heads using the hand methods for application of fungicide, production and inoculation of the fungal spores, and harvesting for analysis that are typically used for small plots *versus* the typical use of farm machinery for fungicide application and harvesting, and the natural source of fungal inoculum. These differences in technology may result in a difference of two to three fold. In this study, the gap between the small plot and large plot/small farm studies was much smaller; the difference in the case of the P125 + T125 treatment was not more than 10%. This means that with careful field technology on the farm scale, we can come much closer to the small plot results at optimum conditions. The significance of the high efficacy of fungicide reduction of FHB in the small plot tests is that this could be considered as a prediction of what can be achieved under field conditions. Although Paul *et al.* (45) concluded that a 20-30% reduction in FHB is not worth the spray, we think that the high efficacies we have seen for the best fungicides raise the hope that we can be much more successful with the best existing fungicides. 

Early studies on the effects of fungicides on FHB measured only the yield of grain at the conclusion of the study. Later on, the measurement of FDK was introduced, but it became apparent that the correlation between FDK and FHB at low infection levels was not close enough to adequately predict marketability of the grain. Now, with the existence of official toxin limits, the level of DON contamination is the most important trait to measure. However, because it is time-consuming and difficult to measure DON levels, it would be desirable to find a close correlation between DON levels and another, easily detectable trait. The data on FHB, FDK values, DON contamination, and yield loss correlated closely in all our tests. The correlation between two traits is generally above 0.90, but closeness up to *r* = 0.97 was also found. When we analyzed the data across years (*n* = 71), the correlations did not change much, although between traits they were normally higher than *r* = 0.80. Even when we checked the 725 data pairs for FDK and DON, the correlation was at *r* = 0.7697. However, the correlation plummeted to *r* = 0.2702 when we checked only the samples having up to 2% FDK, *i.e.*, when the amount of disease was low. We also found samples rated at 1% FDK which had 8 mg kg^−1^ DON. Reports have claimed there is poor correlation between traits [5,63,64]. However, the method of grain evaluation may play a part in discrepancies among reports. Close correlations were normally found [10,27,50,65,66] when all the grains were kept after threshing and not subjected to machine harvesting. We believe that methodical problems may play a significant role in the differences in test results. Most fungicide field tests with artificial and natural inoculation are harvested with a combine with the result that the light, infected grains will be blown out, resulting in a loss of 30–40% of the FDKs (Mesterházy, unpublished). This lessens the correlation between FHB and FDK as well as between FHB and DON [4]. Another reason for low correlation between traits may be a result if there is low infection; in this case the differentiation between fungicides or cultivars cannot be satisfactory. A third reason can be that coverage with fungicides may not be consistent which can therefore produce large differences in fungicide treatment assessments. We think that a critical review of the whole methodology is necessary to improve results in this respect. However, we think we must use the precise methodology in basic studies, otherwise, the conclusions may be false.

While Wilcoxson [4] stressed that FHB control by a fungicide is good when FDK 5%, we now suggest that control is effective when DON contamination is lower than 2 mg kg^−1^ in the USA or 1.25 mg kg^−1^ effect has not been evaluated until now. The set of experiments we conducted allowed us to follow the effect of fungicides in 60 epidemic situations. The statistical analyses we used were borrowed from the plant breeding practice [46,47]. High and significant stability differences were found between fungicides in all traits (FHB, FDK, DON levels). Stability clearly means a fungicide is able to give very good or excellent control in all or nearly all epidemic situations It is important that high stability across ecological conditions can be achieved only with highly effective fungicides. Our use of highly different epidemic conditions was a prerequisite for the evaluation of stability and efficacy. However, under mild epidemic conditions, the fungicide differences were more difficult to discern. 

According to Wale [67] the fungicide effectiveness depends also on curative activity and persistence, *i.e.*, how long the fungicide keeps a protective concentration in plants. According to our data (not published), effective triazoles protect at least three weeks, but the less effective carbendazim protects for only up to two weeks. The time of the last spraying is also of importance. It is clear that in most applications FHB, FDK and DON will be decreased to a similar extent by a fungicide. As the amount of DON is most important in the consideration for food safety, it is imperative to keep these levels low. We have, however, seen cases when fungicide application increased DON contamination. Azoxystrobin increased DON contamination [3,68] in several studies while another study found that suboptimal doses of fungicides increased DON production [31,69]. A new fungicide from China, JS399-19, and belonging to the cyanoacrylate fungicide group [70] appears to be better than carbendazim and further testing with the much more effective tebuconazole and prothioconazole fungicides should be done. 

In these studies, the fungicide effect against *F. graminearum* and *F. culmorum* was actually the same. Simpson *et al.* [71] reported similar activity against *F. culmorum* and *F. avenaceum*, but not against *Microdochium nivale* (syn. *F. nivale*). This means that further studies in the *Fusarium* spp. and fungicide effect may be necessary. 

### 4.2. Influence of Cultivars

The role of cultivar resistance has been mentioned many times in the literature [3,26,29,45,53]. In practice, however, this point is seldom significant as most cultivars are susceptible to FHB and the cultivar resistance differences are moderate [5,72,73,74]. However, as this paper shows, even smaller resistance differences have high significance in the chemical control by fungicides. The more resistant cultivars can be protected more successfully, as the efficacy can be better by 50-60% compared to highly susceptible varieties, but exceptions may be present. Further, an 80% decrease in a susceptible cultivar may still result in 3-4 mg kg^−1^ DON contamination, whereas in a resistant cultivar the DON levels may be reduced to below the official limit value. The better performance of spring wheat in the fungicide tests [26,29,45] may be explained by the faster development of the plants. In a winter wheat, 6-7 weeks may pass between anthesis and ripening, whereas in spring wheat it is 1-2 weeks shorter. So even if the susceptibility is the same, the susceptibility window is shorter. This may explain the excellent results of the moderately resistant Csillag spring wheat in 2010 in Hungary, as all that was raised could be bought because of low toxin contamination, whereas later winter wheat varieties were so contaminated that none could be bought (75). The earliness has another additional advantage, as the fungicide concentration in the plant remains active during the highly susceptible development period. A slower developing cultivar could still be susceptible after the 4 week control by the fungicide. A longer flowering period may also be detrimental as there is no optimum time for control.

Other factors to consider for development of FHB resistant cultivars include the timing of the development of tillers. If the main tillers are out and there are many more in the boot stages, the later developing tillers will not receive fungicide from the early control spray. So, it is best if head development is all at the same time so they will be sprayed with the fungicide at the optimal time. Tall plants tend to have lodging damage [76] and lodged stands are much more exposed to FHB infection as the morning dew stays on lodged plants much longer than on standing plants. Therefore, shorter cultivars are better as they seldom have heavy lodging problems, although lodging does not rely only on height as root systems and other traits are also a factor influencing standability. The canopy structure also influences fungicide treatment. When the head supporting node is long, fungicide coverage can be much better than in cultivars where the heads are just above the flag leaves. Also, some varieties have 2-3 ear levels in the stand. The upper head level has no problem with receiving the fungicide from every side, however the shorter tillers may receive much less fungicide and therefore will have much less protection. We think, therefore, that wheat breeders should consider: early and uniform flowering; good to excellent lodging resistance; development of main and secondary tillers within 1-3 days of each other; ear height should be the same, *i.e.*, all heads should take place within 20 cm vertical distance; heads should be at least 15 cm above flag leaves; and erect leaves should be avoided if possible as they may make a shadow against fungicide spray. In addition, physiological head blight resistance is also urgently needed. 

### 4.3. Management Inputs

The method of application of fungicides is extremely important and reports of poor FHB control by fungicides is most likely due to the poor coverage of the heads with the fungicides being tested [3,41,42,61,77,78]. However, it should be noted that when using the most effective fungicides, we found that FHB was reduced around 70-80% regardless of whether the TeeJet XR or Turbo FloodJet nozzles were used. However, the data shows that the reduction was consistently greater with the Turbo FloodJet. On average, full coverage significantly increases the fungicide efficacy. We therefore believe that without updated spraying technology, the chemical control of FHB cannot be resolved, even if more effective fungicides are developed. Because we found that the most effective fungicides produced a more than 80% reduction in FHB in farm scale tests, which was comparable to the small plot results, this means that the technology gap could be decreased to about 10-15%. 

The assumption is that minimal or no translocation of the fungicides occurs between ears and leaves [35], but perhaps more studies should be done. The data from this paper support the view that good coverage may increase FHB control significantly, but can never transform a weak fungicide to an excellent one. In our tests, the Turbo FloodJet nozzle gave superior results over the traditional TeeJet XR nozzle resulting in as much as 98% reduction in FHB when using P125 + T125. This is far more than the 20–30% of the everyday practice that has been previously reported. Other management inputs should also be investigated, such as timing of the application, the above mentioned variety influences, environmental factors such as rain, and lastly, cost of application.

It is common knowledge that following corn in crop rotation , seriously increases the probability of FHB epidemics in wheat. Plowing under the corn debris is therefore important, and should be followed by a highly effective fungicide treatment as a preventative measure. The more susceptible cultivars may be successfully grown after previous crops such as soybean, canola or other crops, although it is recommended that highly susceptible cultivars should be withdrawn from production as they cannot be protected effectively under heavy FHB epidemic conditions. It would be best, therefore, before registration of a wheat variety that a FHB resistance test be performed in order to diminish FHB epidemics.

### 4.4. Conclusions

It is clear that the problem of fungicide application is far more than a decision to spray or not to spray. The plan should include decisions about the variety of wheat (spring/winter), the cultivar (FHB resistance), tillage (no or only minimum plant residue), crop rotation (maize, wheat, soybean as prior crop), brand of fungicide (effectiveness), time of fungicide application, type of nozzle to use, moisture/temperature conditions, and cost of application. Disease forecasting models, such as the Michigan State Univ. (79) which is based on cultivar susceptibility, flowering time, and weather, and the *Fusarium* Head Blight Risk Assessment Tool (80) (both available for the USA) should help in the decision-making process. With the many factors involved in the decision of fungicide control, cultivar specific plant protection programs should be developed. The idea is not new [27,81], but should be updated with the increase in knowledge. The technology gap could be narrowed significantly, making fungicide treatments of wheat more effective and economical with the expected result of meeting the food and feed safety standards of today and the future. 

## Ackonwledgements

The authors would like to express their thanks to OMFB (grants 6315 and 6777), OTKA (TS 040887 and D 38486), NKFP (Wheat Consortium 4/038/2001), FVM (21a/2000 and grant for Technology Improvement), EU FP5 Fucomyr (QLK5-CT-2001-02044), EU FP7 MycoRed (KBBE-2007-2-5-05). The authors are grateful to the fungicide producers for providing fungicides. The finishing work was supported by the Deak Zrt. project (2009-2011). The authors are indebted to Nancy Alexander for her innovative ideas for improving and editing the manuscript.
